# P29 Catheter-associated urinary tract infections, potential for diagnostic and antimicrobial stewardship

**DOI:** 10.1093/jacamr/dlad077.033

**Published:** 2023-08-02

**Authors:** Siobháin Kelly, Sinead O’ Donnell, Ailish Powderly, Fidelma Fitzpatrick

**Affiliations:** Department of Clinical Microbiology, Beaumont Hospital, Dublin, Ireland; Department of Clinical Microbiology, Beaumont Hospital, Dublin, Ireland; Department of Clinical Microbiology, The Royal College of Surgeons in Ireland (RCSI), Ireland; Department of Clinical Microbiology, Beaumont Hospital, Dublin, Ireland; Department of Clinical Microbiology, Beaumont Hospital, Dublin, Ireland; Department of Clinical Microbiology, The Royal College of Surgeons in Ireland (RCSI), Ireland

## Abstract

**Background and Objectives:**

Catheter-associated urinary tract infections (CAUTIs) are one of the most common healthcare-associated infections.^1^ Microbiological investigation of catheter specimens of urine (CSUs) results in a significant workload for microbiology laboratories. As bacteria cultured from CSUs often reflect colonization rather than infection, we must prudently interpret these culture results and judiciously prescribe antimicrobials in this setting. For these reasons, we felt it was important to assess if CAUTIs are being appropriately investigated and managed in our hospital.

**Methods:**

We identified all the CSUs received in the microbiology laboratory from inpatients over a 7 day period in an 820-bed adult tertiary referral hospital. Once identified, a retrospective review of each patient’s clinical and drug charts was undertaken. The CDC definition of CAUTI was applied for the purpose of this audit.^2^

**Results:**

A total of 126 CSUs from 101 patients were included. The majority of patients (*n*=79, 78%) had a urinary catheter introduced during their hospital admission. At the time of CSU sampling, suspicion of infection was documented in only 47 patients. Only 9% of patients who had a CSU processed met the criteria for a CAUTI.^2^ Fifty-six percent of patients were commenced on antibiotics, despite only 31% returning a culture positive CSU sample. CSU results were documented in only 10% of patient charts. In cases where a CAUTI was suspected, it took an average of 6.75 days for the urinary catheter to be exchanged or removed. Four patients continued to receive an antibiotic despite the organism isolated being reported as resistant.

**Conclusions:**

Overall, we noted that there was poor documentation of both urinary catheter status and of CSU results. Our audit revealed that several patients had multiple specimens processed within the 7 day period. Most patients had no clear indication for CSU sampling. From an antimicrobial stewardship perspective, there was unnecessary and inappropriate prescribing of antimicrobials. The results of this audit show that there is potential for both diagnostic and antimicrobial stewardship in the management of CAUTIs.
